# A novel task of canine olfaction for use in adult and senior pet dogs

**DOI:** 10.1038/s41598-023-29361-x

**Published:** 2023-02-08

**Authors:** Michael Z. Khan, Alejandra Mondino, Katharine Russell, Beth Case, Gilad Fefer, Hope Woods, Natasha Olby, Margaret Gruen

**Affiliations:** Department of Clinical Sciences, College of Veterinary Medicine, North Carolina State University, Raleigh, NC 27607 USA

**Keywords:** Cognitive ageing, Neural ageing, Olfactory system, Sensory processing

## Abstract

While much work has been done in the field of canine olfaction, there has been little exploration of hyposmia or anosmia. This is partly due to difficulties in reducing confounds like training history and environmental distraction. The current study describes a novel olfaction test using spontaneous search behavior in dogs to find a hidden food treat in a three-choice task with both light-phase and dark-phase conditions. The study was performed in 18 adult control dogs, 18 senior/geriatric dogs enrolled in a longitudinal aging study, and a single dog with severe nasal pathology. In the senior/geriatric and control groups, dogs performed with higher accuracy (p < 0.0001) and were less likely to show biased selection strategy (p < 0.01) in the dark-phase than light-phase. While senior/geriatric dogs performed above chance, they had lower accuracy in the dark-phase compared to controls (p = 0.036). Dogs who scored higher on an owner questionnaire of cognitive decline showed a positive correlation with performance in the dark-phase; performance on additional cognitive tests did not correlate with performance in the dark-phase. This task can be used to quantify canine olfaction using clearly defined endpoints and spontaneous behaviors thus making it feasible to compare between and within groups of pet dogs.

## Introduction

There is no doubt that dogs’ remarkable sense of smell is one of their defining features. It is well documented that dogs can be trained to discriminate between and locate an array of biological and chemical olfactory cues^1^, leading to their inclusion in a variety of working environments. Dogs show remarkable ability in detecting trace volatile compounds, with evidence of detecting unique odorants in the range of parts per billion to parts per trillion^2^. In order to identify, analyze, and locate the source of an odorant, dogs engage in conscious sniffing behaviors^3,4^. Sniffing begins by forcing air containing odorants through the nostrils to the olfactory epithelium^5^. The individual odorants activate the olfactory receptor cells within the olfactory epithelium^6^. These cells transmit their signals directly to the olfactory bulb in the ipsilateral brain hemisphere for pattern recognition thus creating a unique odor. The odor information is then transmitted to various parts of the brain for memory formation, emotional response, and cortical processing^4,7–9^.

Despite multiple studies of canine olfaction, very few characterize hyposmia (reduced sense of smell) or anosmia (absent sense of smell)^10^. Certain medications, viral infections, and radiotherapy have been linked with diminished sense of smell or altered taste perception in humans^11–13^, but no comparative studies exist for dogs. This is due, in part, to the lack of a functional test with clear endpoints and confidence that dogs are using their sense of smell rather than visual cues to make choices. Previous studies in olfaction with companion (pet) dogs primarily used one of two techniques. The first technique involves operant conditioning in which the dog is trained to give a signal (i.e., laying down, barking, or pawing) when presented with a paired odorant. The concentration of the odorant is then increased or decreased to test the limit at which the dog still responds^14^. In addition to a functional olfactory system, dogs’ performance of this task requires them to have the cognitive capacity to quickly form new associations and be sufficiently motivated to perform the requested signal^15–17^. The second technique attempts to address the need for learning by evaluating spontaneous behaviors using inherently rewarding odorants (often from food). For example, investigators may hide a food treat within a testing arena and record the time it takes the dog to locate the treat^18–21^; this method may be influenced by search patterns, visual environmental cues, and movement speed, making inter-dog comparisons difficult.

With both operant and spontaneous testing techniques there are concerns that visual stimuli, other environmental odorants, variability of spontaneous behavior, or clues from the experimenter are impacting the observed results^19,21–24^. Several studies have shown that in two-choice tasks (where a food treat is hidden under an opaque, non-airtight container, in one of two locations), during the control conditions dogs will consistently perform at chance when no external cue (such as pointing to the correct location) is provided^25–29^. This demonstrates when visual cues are present untrained dogs may preferentially use these first. There exist important gaps in the knowledge base that can be addressed with a functional olfaction test specifically targeting dogs with diminished or absent sense of smell^20^.

Development of a functional test is also of importance as previous research in humans has identified hyposmia as a predictor of cognitive decline^30^. A diminished sense of smell has also been correlated with an increased depressive state in both humans and mice^31,32^; no such study has been performed with dogs. In dogs, there is evidence that histopathologic changes to the olfactory epithelium occur with aging^33^, and surveys have suggested an owner perception of olfaction and other sensory decline in aging pet dogs^34^. Previous studies have also shown a decline in performance during a food searching task in dogs with cognitive impairment^18,35^, however these tasks were performed under conditions where visual cues may factor into dogs’ decision-making process.

Here we present an alternative method for testing canine olfaction that employs a spontaneous, untrained response from the dog and uses a dark-phase to remove visual cues from the testing environment, requiring the dog to rely on their sense of smell to reach the food reward. Our study has four separate aims to demonstrate the use of this task. First, we aim to create a quantitative scale that could be used to detect baseline or diminished olfaction in any population of dog. Second, by comparing the same task in light-phase and dark-phase conditions we aim to demonstrate the impact of visual bias on choice selection in untrained dogs. Given dogs’ propensity to use visual cues when available, we predict that dogs performing the food search task will show decreased performance in the presence of visual stimuli (light-phase) compared to the dark-phase. Third, if healthy adult dogs are able to demonstrate proficiency at the task, we further predict that they will perform better than senior/geriatric dogs, and that dogs with nasal pathology will have severely reduced performance at the task. Fourth, within the senior/geriatric population, we also aim to explore the effects of cognitive decline, measured via cognitive testing and owner questionnaires, on performance in the task.

## Materials and methods

### Study population

Dogs in this prospective study were recruited as two separate groups of healthy adult (control) (n = 18) and senior/geriatric dogs (n = 18) (Table 1). As a proof-of-principle, a single dog (n = 1) with suspected anosmia (secondary to inflammatory sinusitis and nasal radiotherapy) was also enrolled. Control dogs were recruited via email survey from the students and staff at the North Carolina State University College of Veterinary Medicine (NCSU-CVM) and were included in the study if they were adult (2–7 years old), had no owner reported history of significant medical illness and no significant abnormalities found by physical and orthopedic exams performed by a veterinarian. Dogs were excluded if they previously showed signs of aggression over food, had dietary restrictions, formal nose-work training, or showed anxiety around unfamiliar people that would preclude their ability to participate in the task.

**Table 1:** Summary demographic information for the study dogs. Fractional life span was not calculated for the control group or the nasal pathology case and are not reported in this dataset. Additionally CADES score was not evaluated in the control group but was evaluated in the nasal pathology case due to his age at the time of testing.

Group	n	Age range (mean ± SD)	Sex (MC/FS)	Fractional lifespan ratio (mean ± SD)	CADES score range (mean ± SD)
Control	18	2–6.5 years (4.6 years ± 1.21)	9/9	–	–
Senior/geriatric	18	10.8–14.8 years (13 years ± 1.63)	7/11	0.81–1.14 (1.02 ± 0.097)	0–50 (21 ± 15.2)
Nasal pathology	1	10 years	1/0	–	0

Senior/geriatric dogs were recruited from a population participating in a longitudinal study of neuro-aging at NCSU-CVM^36^. These dogs were at or beyond the last 25% of their expected lifespan as normalized by the American Kennel Club breed standards. Fractional life span was also calculated by comparing the ratio of their age to their calculated expected life span [13.62 + (0.0702 $\times$ H) − (0.0538 $\times$ W)]^37^ where H = height in inches from the floor to the withers and W = weight in pounds. Dogs were in varying stages of cognitive decline as assessed by the Canine Dementia Scale (CADES)^38^ which was completed by owners within 1 week of participating in the study. This scale asks owners to quantify the frequency of common signs of cognitive dysfunction, with a higher overall score indicating more severe cognitive impairment.

All owners were informed of the details of the study and signed a consent form before their dog participated in any experiment, and dogs remained housed by their owners before and after their visit to the NCSU-CVM. All procedures were approved by the North Carolina State University Institutional Animal Care and Use Committee, were carried out in accordance with relevant guidelines and regulations.

### Food search trials

**Figure 1:**
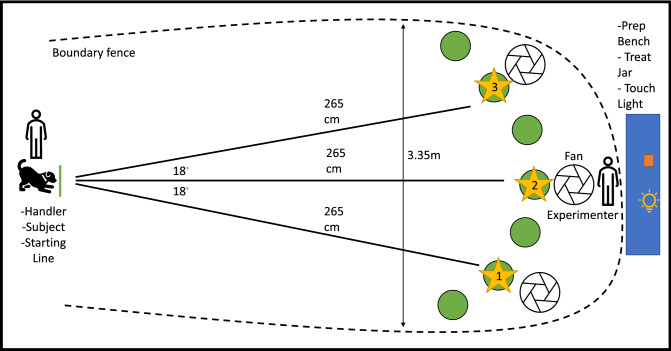
Experimental set up for the olfaction test. The dog is positioned behind the starting line before the trial begins. Phosphorescent dots are placed on the green circles to designate the treat locations and provide guides on where to place the treats in the dark-phase. Fans are angled toward the starting line behind the treat containers. The experimenter collects a treat from the workbench area before sealing the container and walking to the starting line to present the treat to the dog. The experimenter places the treat in a designated open container on the workbench then places the baited and sham containers in their designated positions, moving from position 1 to position 3 (designated by star symbols).

The experiment was conducted in a dedicated cognitive testing space at NCSU-CVM. The room was 5 m $\times$ 4.5 m and surrounded by four cinderblock walls in a quiet area of the Health and Wellness Center. The testing arena was defined by a wire fence 3.35 m across (Fig. 1). A video example of the test is available in the supplementary materials (informed consent was obtained from handler in the Nasal Pathology Trial video for publication of identifying information/images in an online open-access publication). Before dogs entered the space, the floor was cleaned with a mop and mild detergent then allowed to dry. All trash cans were removed from the room before testing began. Two white noise machines (Yogasleep, Dohm Classic, USA), were turned on inside the cognitive testing room and outside the room door to reduce the amount of extraneous auditory stimuli. The dogs were given a 10-min acclimation period in the testing area where they were allowed free exploration of the room and interaction with the investigators. Testing trials were recorded from an overhead angled view using an infrared camera set to view the whole arena (Amcrest 1080P pan/tilt wifi-camera, USA). A blackout curtain was placed over the doorframe to ensure no additional light was present during the dark-phase trials. Seven green, phosphorescent markers (1 cm × 1 cm) were placed 265 cm equidistant from the starting line (also denoted by a phosphorescent marker 3.5 cm long) and 17 cm from each other. The phosphorescent markers were placed to help the experimenter navigate during the dark-phase condition and during placement of the treat rewards. These markers emitted spot phosphorescence and did not appear to change the overall visibility in the room. Three 4-speed, 6 inch diameter fans (iHoven, KF4) operating at the lowest setting were placed over markers two, four, and six and angled toward the center of the start line, which will henceforth be referred to as treat positions one, two, and three, respectively.

To start the test, dogs stood or sat behind the starting line held on a short leash by the handler. The leash had a 5 cm × 1 cm piece of phosphorescent tape to allow the handler to find the dog in the dark. The experimenter extracted a 1 cm piece of Pup-Peroni^®^ from a sealed container and presented it to the dog using forceps to avoid cross-contamination of odorants on their hands. With their back turned to the dog, the experimenter placed the treat in a designated paper bowl (positive bowl) against the rim of the bowl to prevent it from being visible until approached. The experimenter picked up the positive bowl and two identical but empty negative bowls. The bowls were placed on treat position one, two, and three directly in front of the fans. The position of the positive bowl and negative bowls were determined by a set pseudo-randomized pattern. Each positive bowl position was represented twice over each set of six trials. The experimenter always placed bowls in sequence from position one, to position two, to position three (left-to-right). Once all bowls were placed the experimenter would return behind the middle bowl and say, “Okay” and the dog would be released by the handler from the starting position. The trial was ended once the dog found the treat or ninety seconds had passed. If a dog did not locate the treat within ninety seconds, they were shown the position of the treat and allowed to obtain the reward before moving on to the next trial.

After six trials in light-phase, the overhead lights were turned off and a small touch light was turned on for the experimenter to see while preparing the bowls for the next trial. The same procedure and pattern were followed during this dark-phase except that after the treat was shown to the dog and placed in a container, the touch light was turned off and bowls were placed while the only light in the room was from the small point luminescence markers on the floor and leash.

After six trials in the dark-phase, a second six-trial light-phase was repeated using a different bowl placement pattern with each positive position represented twice. Finally, a second dark-phase trial was performed using the same pattern as the second light-phase. Both the second set in the light and dark phase were done to ensure continued motivation throughout the trials, and to demonstrate problem solving strategies remained consistent between both phases.

### Video scoring

Results from each trial were scored from video using Behavioral Observation Research Interactive Software (BORIS v. 7.12.12; University of Torino, Torino, Italy). Each trial was scored independently for correct choice on first selection (binary), time to first choice (milliseconds), and to correct choice (milliseconds).

A choice was defined as the dog breaking the vertical plane of the bowl with their head held lower than their shoulders. A correct choice was defined as choosing the positive bowl containing the treat. Time measurements were started when the experimenter said “Okay” and ended when the defined choice was made. To compare time to correct choice between individual dogs of different sizes and with different movement speeds, we used correct choice time normalized by individual light-phase mean time to the first choice over the 12 trials. The mean time to first choice in the light phase was used for the movement correction due to the direct path most dogs took to the first choice in the light.

### Cognitive testing

Additional cognitive tests were performed for the senior/geriatric population of dogs as described in Fefer et al.^36^. Cognitive tests included sustained gaze, working memory, inhibitory control, and spatial detour. Briefly, sustained gaze required the dog to maintain eye contact with the experimenter holding a treat and was measured in seconds. Working memory was assessed at the highest threshold (seconds) the dog could remember the location of a treat in a two-choice task following an increasing delay. Inhibitory control was measured as percent of correct interactions with a clear cylinder (defined as not touching the cylinder while obtaining a treat). Spatial detour used the same cylinder and success criteria as inhibitory control, but a barrier was placed on the dog’s preferred side of entry. See supplementary material for full descriptions and videos of these cognitive tests.

### Statistical analysis

Statistical analyses were performed using JMP Pro 16.0.0 (SAS Institute, Cary, NC). As most dogs would walk straight to their first choice during the light-phase, time to the correct choice was modified to correct for individual walking speed by dividing the average time to correct choice in either phase by the average time to the first choice across both light-phases.

Number of correct trials was analyzed independently for each group. A score of 4/12 (33.3%) correct would define the average selection by chance with a score of ≥ 8/12 (≥ 66.7%) representing a score significantly greater than chance (p = 0.02, based on binomial distribution).

Choice selection biases were also determined by categorizing the dogs into 3 search patterns: positional, recency, and random. Positional bias was defined as the dog choosing a single position regardless of treat location in at least 8/12 (p = 0.02) of trials. Recency bias was defined as choosing where the treat was on the previous trial for 6/11 (p = 0.04) of choices (and if this bias was shown a greater percentage of times than positional bias). Recency bias was calculated out of 11 trials as there no preceding position on trial 1. If neither bias was detected the dogs were categorized as not showing a bias. Based on the positive bowl position pattern, the positional bias and recency bias would score respectively 4/12 and 3/11 correct if the dog solely used either of these searching strategies.

Light-phase and dark-phase comparisons of percent correct and time to correct choice were analyzed using matched paired analysis (Wilcoxon Signed Ranked test). A Bowker’s test of agreement was used to compare bias formation in both the light and dark conditions. Comparisons between senior/geriatric and control groups were made using a one-way ANOVA and pooled t-test with a Bonferroni-corrected p-value of 0.013 for the 4 comparisons made between the groups. Analyses of age, fractional lifespan, CADES score, and cognitive test performance were done using multivariate analysis and a nonparametric Spearman’s ρ test. For multivariate analysis the Bonferroni-corrected p-value to determine significance was set at 0.006 as 8 comparisons were made within the senior/geriatric group. The dog undergoing radiation therapy was not included in statistical analysis due to his inclusion as proof-of-principle.

## Results

### Subjects

Demographic details from included dogs are available in the supplementary materials, with summary details in Table 1. Breeds represented in the control group included: mixed breed dogs (12), Pembroke Welsh Corgi (1), Shetland Sheepdog (1), Scottish Terrier (1), German Shepherd (1), Border Collie (1), Beagle (1). Breeds represented in the aging group included: mixed breed dogs (6), Siberian Husky (1), Pomeranian (1), Jack Russell Terrier (1), Irish Setter (1), Golden Retriever (3), German Shorthair Pointer (1), Dachshund (1), Brittany Spaniel (1), Basset Hound (1), American Staffordshire Terrier (1). The one nasal pathology case was also a Beagle.

### Light-phase vs. dark-phase trials in the control group

As percentage of correct choices was not significantly different between the first and second light and dark phase (Wilcoxon Signed Rank, Light: S = 6, p = 0.69. Dark: S = − 19, p = 0.57), these were combined for remaining analyses. The control group had significantly more correct responses during the pooled dark-phase trials compared to the light-phase trials (Wilcoxon Signed Rank, S = 85.5, p < 0.0001). In the light-phase, the average percent correct for the control group was 47.2% (± 10.7%) with 2 of 18 dogs displaying a correct choice selection above chance (≥ 66.7%). In the dark-phase, the average percent correct for the control group was 79.2% (± 14.3) with 16/18 dogs displaying a correct choice selection above chance (≥ 66.7%).

Additionally, the control group showed a longer latency (corrected for movement speed) to the correct choice in the dark-phase condition (mean ± SD = 3.18 a.u. ± 1.16) compared to the light-phase condition (mean ± SD = 1.69 a.u. ± 0.30) (Wilcoxon Signed Rank, S = − 76.5, p < 0.0001).

Dogs in the control group were significantly more likely to display a selection bias in the light condition (n = 12) compared to the dark (n = 2) ($\chi$^2^ = 10, p < 0.01). The most frequent bias was positional selection, with position 2 (n = 5) and position 3 (n = 5) in the light-phase and position 2 in the dark-phase condition (n = 2); in the light-phase, the remaining two dogs showed recency bias.

### Senior/geriatric vs. control

The senior/geriatric group also showed no difference in percent correct between the first and second light and dark phase (Wilcoxon Signed Rank, Light: S = 13.5, p = 0.46. Dark: S = − 3, p = 0.83). As such their results were pooled for the remainder of the analyses. The senior/geriatric group scored significantly more correct responses in the dark-phase condition compared to the light-phase (S = 74.5, p < 0.001). The control group scored significantly higher than the senior/geriatric group for percent correct during the dark-phase condition ((t_(34)_ = 2.18, p = 0.04), Table 2), however this was not significant if the threshold p-value was adjusted for multiple comparisons (threshold p = 0.013). There was also no significant difference between groups in the number of dogs above chance in the dark-phase ($\chi$^2^ = 1.60, p = 0.21). In the light-phase condition, there was no group difference in percent correct. There was no significant difference between senior/geriatric and control groups in the latency to first choice in the light condition (t_(33)_ = − 1.07, p = 0.29). When correcting for movement speed the control group located the correct choice significantly more quickly in the dark-phase than the senior/geriatric group (Table 2). There was no significant difference between groups in formation of a side bias in the light-phase ($\chi$^2^ = 0.55, p = 0.46) or dark phase ($\chi$^2^ = 0.8, p = 0.37).

**Table 2:** Comparison between the control and senior/geriatric groups in both the light and dark-phase conditions. Comparisons to the control group are reported with t-statistic and relevant p-value. *Indicates a p-value of less than 0.013 (Bonferroni corrected p-value for 4 comparisons).

	Control group Mean ± SD	N	Senior/geriatric group Mean ± SD	N	Test statistic (compared to control group)	p-value
% Correct in light-phase	47.2 ± 10.7	18	41.2 ± 13.54	18	t_(34)_ = 1.48	0.14
% Correct in dark-phase	79.2 ± 14.3	18	66.7 ± 19.60	18	t_(34)_ = 2.18	0.04
Time to correct choice in light phase (a.u.)	1.69 ± 0.30	17	2.38 ± 1.12	18	t_(33)_ = − 2.47	0.02
Time to correct choice in dark phase (a.u.)	3.18 ± 1.16	17	5.18 ± 2.21	18	t_(33)_ = − 3.11	0.002*

### Variables in the senior/geriatric group

Within the senior/geriatric group, there was a trend toward a significant positive correlation between absolute age and percent correct in the dark-phase (ρ = 0.58, p = 0.01). However, this correlation was weaker and not statistically significant when using fractional lifespan ratio (ρ = 0.30, p = 0.24). There was a trend toward a statistically significant positive correlation between percent correct in the dark-phase and total CADES score (ρ = 0.52, p = 0.03). None of the cognitive testing outcomes measured were significantly correlated with accuracy in the dark-phase (Table 3).

**Table 3:** Multivariate analysis of cognitive tests and CADES scores in the senior/geriatric group of dogs compared to their percent of correct choices in the dark-phase. Nonparametric Spearman’s ρ are reported along with associated p-value. Significance is set at 0.006 to account for multiple comparsions.

Variable	By variable	Spearman ρ	Prob >|ρ|
% Correct dark-phase	Age	0.58	0.01
	Fractional lifespan ratio	0.30	0.24
	Sustained gaze (s)	0.31	0.31
	Working memory (s)	− 0.08	0.80
	Inhibitory control (%Correct)	0.03	0.89
	Detour (%Correct)	− 0.35	0.16
	CADES	0.52	0.03
	% Correct light	− 0.11	0.66

### Nasal pathology proof of concept

A single dog was recruited from the radiation oncology department at North Carolina State University who had previously been diagnosed with mild to moderate chronic lymphoplasmacytic and neutrophilic rhinitis and had undergone targeted radiation therapy of the entire nasal cavity and associated sinuses approximately one year prior to testing. The dog’s owners were suspicious of anosmia due to the dog’s inability to locate treats unless shown at home.

This dog was correct in 58% of trials in the light-phase, but only correct in 16.7% of trials in the dark-phase. Of note, during the dark phase when the dog would “choose” the correct bowl placement, the dog often did not eat the treat inside the bowl until the touch light illuminated the room (see supplementary material for video of representative trials).

## Discussion

Our results demonstrate development of a test of canine olfaction that has a quantitative scale with definitive endpoints, requires dogs to depend solely on their sense of smell rather than vision, allows for a spontaneous, untrained behavior to reduce the impact of impaired learning or memory capacity. In addition, we were able to show a trend of diminished sense of smell that can be assessed longitudinally or between groups of dogs.

### Impact of visual environmental stimuli

We found a significant difference between dogs’ performance in the light-phase compared to their performance in the dark-phase. The percent of correct choices clearly showed an improvement once visual stimuli were reduced. Our findings are consistent with previous research showing that untrained dogs will make decisions based on visual stimuli in a two-choice task^25–29^. While surprising given dogs’ well-developed sense of smell, this could be a result of visual processing occurring at a higher speed than olfactory processing, which depends on the respiration or sniff cycle^3,7,39^. While dogs did show an increased latency to find the correct treat location in the dark, this is likely indicative of dogs choosing to navigate the arena with a different sense other than sight. We attempted to normalize the data to the dog’s walking speed using the latency to first choice in the light as the dogs basic movement speed. However, comparisons between latency to find the treat could prove challenging as several confounding variables are not accounted for with this test such as spontaneous search pattern differences. While many tests of canine olfaction have used time to find a treat as a dependent measure, we argue percent correct is a stronger measurement in the untrained dog. Spontaneous search patterns and motivation to find a treat may influence the dog’s path and ultimately time to the treat whereas the percent correct will remain unaffected regardless of search pattern so long as the dog stays sufficiently motivated to engage in the task.

In addition to the improvement in accuracy, dogs were also less likely to display a biased selection strategy in the dark-phase. This further supports that in the light-phase, without clear cues from the environment or the experimenter as to the correct choice, dogs adopt a visual choice strategy rather than use their sense of smell. Many dogs showed a positional bias by selecting either the bowl directly in front of the experimenter, or the last bowl the experimenter placed. When bias was observed in the dark-phase condition, the bowl in front of the experimenter was chosen most frequently. Previous research has described lateralization of odorant and odor processing in the canine nostril and brain^40,41^. While these studies found that dogs will preferentially sniff with the left nostril for familiar odorants^6^, in the current study this bias occurred infrequently in the dark-phase and we argue that the more likely cause is the experimenter placing the bowl down last as a visual cue for the dog in the light-phase. This theory aligns with previous research that suggest the presence or path of the experimenter remains a larger influence or a source of information for the dog in olfaction testing^42^.

By removing as many visual stimuli as possible we were able to minimize the confounding variables that often arise when studying canine olfaction. Validation for the test is further demonstrated in our proof-of-principle. This dog still performed the task in the light-phase and dark-phase conditions, however, their performance in the dark-phase was dramatically reduced compared to either the control or aging population. When this dog “chose” the correct position in the dark it was often by stepping into the bowl and they would not retrieve the treat until the small touch light was turned on to illuminate the bowl; this indicated that visual perception was essential for this dog to complete the task. We explored other ways to validate the test using temporary induced clinical anosmia, however chemical or physical means of producing anosmia are often invasive and have potentially irreversible side effects^43^. Previous studies have also suggested medically induced hyposomnia in dogs is possible through steroid injection^44^ or suspected after canine parainfluenza virus as measured through electroencephalography^45^, however these were not pursued. Dogs who were trained in olfaction work (“nosework”) were also included in an earlier iteration of the protocol, however while the trained dogs were faster in making their selections, no difference in accuracy between the trained dogs and untrained dogs was observed in that pilot work. Several changes to the testing setup were made between the pilot study and the current protocol, and revisiting the trained dogs may prove useful in exploring the upper bounds of this test. However, since trained dogs need to be trained to a specific scent and often cued to start exploration, this may be a confounding variable for assessing the spontaneous behavior utilized in our test.

### Limitations in development of the task

Multiple considerations were taken to remove as many environmental stimuli as possible during this test including using a white noise machine, a mild detergent to clean in between dogs, maintaining a consistent room temperature, and removing other treats or potential strong odorant sources; however there are undoubtedly several other factors that influence the delivery or processing of the odorant such as humidity, human scents, and other animal scents that may linger despite cleaning^6,24,46^. One improvement for future studies could be to remove both the experimenter and handler from the room before starting the task. This was not done in the current study to minimize walking back and forth across the scent trail once the food was placed. Although the experimenter stood behind the fans this could account for some of the side bias formation at position 2 observed during the dark-phase trials.

Further validation of the protocol could be done using additional dogs with varying degrees of hyposmia and anosmia. As our proof-of-concept group only contained a single dog with suspected anosmia, additional subjects are needed to increase the strength and confidence of the observed results.

### Potential impact of breed on observed results

Breed differences were not considered in this study but would be of interest in future work. The current literature provides mixed information as to the influence of breed on performance in tests of olfaction. Breed differences were observed in an operant response to varying levels of odorant permeability^20^; dogs traditionally bred for tracking (bloodhounds and beagles) performed better at the task than those who were not (greyhounds). Our study included four beagles and hounds (basset hound and bloodhound mix) with two in each testing group (control and senior/geriatric). The two in the senior/geriatric group performed better than the mean for their group at 75% and 83% correct whereas in the control group one dog performed at the mean (75% correct) and one was above (92% correct). Other work comparing breeds during an operantly trained olfactory task found that pugs outperformed German shepherds, while greyhounds were unable to complete the operant task^16^. In our study, only one German shepherd was included in the control group; while this dog performed above the mean for the group (83% correct) no conclusions can be drawn from this single individual. While no obvious signs of breed differences were shown in our study, this will need to be evaluated in future work and can be investigated using this protocol.

### Effects of aging on olfaction

Our study also demonstrated a trend in reduced capacity for olfaction in senior/geriatric dogs compared to younger ones. This supports previous findings in canine aging and olfaction research^18,35^. As these studies have used time to find a treat or total treats found in the trial as dependent measures, these may be impacted by the search pattern (i.e., air-sniffing vs. ground sniffing) or motivation to find the reward^42^. Our study eliminates visual cues and thus rewards any search strategy that follows an odorant gradient, providing a strong measure of true olfaction capability in senior/geriatric dogs. Further, dogs could perform the task even when experiencing cognitive decline. Dogs with high scores (higher impairment) on owner-completed questionnaires were still able to complete the task at a high level of accuracy. In fact, there was a weak trend showing a higher CADES score correlated with a higher score on the olfaction test, contrary to what we would predict. However, this will need to be evaluated fully in future work as the low number of dogs in the moderate to severe cognitive impairment categories among our study population may have affected our findings. Performance on the olfaction test also did not correlate with our behavioral measures of cognitive impairment. These findings, as well as performance over longitudinal testing, will be evaluated in our future work. While absolute age was associated with poorer performance on this task, when correcting for expected lifespan there did not appear to be any significant correlation. For a better understanding of how age is affecting the dog’s sense of smell, the dogs could further be divided into multiple categories based on their expected life span ratio, however; this would require a much larger population than in the present study.

## Conclusions

Our study demonstrates a novel olfaction test that reduces the impact of environmental stimuli by removing as many visual cues as possible. This test uses spontaneous behaviors from the subject thus removing the need for a training or acquisition period, and includes clear endpoints for evaluation. Using a spontaneous behavior, this test reduces the influence of other cognitive measures and relies primarily subject’s sense of smell. This protocol may have value in the field of canine detection work to identify dogs with high performance, particularly using decreasing concentrations of presented odorant. The capability of the test to detect reduced olfaction capability highlights its potential as a useful measure in the field of canine cognition and aging.

## Supplementary Information

Supplementary Video 1.

Supplementary Video 2.

Supplementary Information.

## Data Availability

The datasets are available from the corresponding author on reasonable request.
