# Supplementary material for: A novel task of canine olfaction for use in adult and senior pet dogs
Source: Sci Rep. 2023 Feb 8;13:2224. doi: 10.1038/s41598-023-29361-x (PMC9908929; doi:10.1038/s41598-023-29361-x)
Supplement: Supplementary file 3 — Supplementary Information. [file 41598_2023_29361_MOESM3_ESM.pdf]

A novel task of canine olfaction for use in adult and senior pet dogs

Michael Z. Khan, Alejandra Mondino, Katharine Russell, Beth Case, Gilad Fefer, Hope Woods, Natasha Olby, Margaret Gruen

Supplementary Table 1. Demographics of participating dogs in the control and senior/aging group. Calculated lifespan and fractional lifespan ratio are shown for senior/aging dogs. Breed and AKC reported lifespan for that breed are provided for purebred dogs.

Study ID	Group	Sex	Age (y)	Weight kg (lbs)	Calculated Lifespan	Fractional Lifespan Ratio	Breed (AKC Reported Life Span)
C1	Control	MC	4.5	21.5 (47.3)			Mixed breed
C2	Control	FS	5.5	12.2 (26.8)			Mixed breed
C3	Control	MC	6.5	13.7 (30.1)			Pembroke Welch Corgi (12-13y)
C4	Control	FS	3	23.8 (52.4)			Mixed breed
C5	Control	MC	3	29.2 (64.2)			Mixed breed
C6	Control	MC	6.5	26.4 (58.1)			Mixed breed
C7	Control	MC	5	9.4 (20.68)			Beagle (10-15y)
C8	Control	MC	6	18.9 (41.58)			Mixed breed
C9	Control	FS	6.5	10 (22.0)			Scottish Terrier (12y)
C10	Control	FS	3	22.8 (50.2)			Mixed breed
C11	Control	MC	5	12.6 (27.7)			Mixed breed
C12	Control	MC	4.5	22.4 (49.3)			Border Collie (12-15y)
C13	Control	FS	5	15.4 (33.9)			Mixed breed
C14	Control	FS	2	-			Mixed breed
C15	Control	MC	6.5	36.2 (80.0)			Mixed breed
C16	Control	FS	2.5	10.8 (23.8)			Shetland Sheepdog (12-14y)
C17	Control	FS	6	32.5 (71.5)			German Shepherd (7-10y)
C18	Control	FS	2	27.5 (60.5)			Mixed breed
S1	Senior/ Geriatric	FS	13.7	24.5 (53.9)	12.21	1.12	Golden retriever (10-12y)
S2	Senior/ Geriatric	MC	10.8	8.6 (18.9)	13.33	0.81	Dachshund (12-16y)
S3	Senior/ Geriatric	FS	14.8	16.2 (35.6)	12.94	1.14	Mixed Breed
S4	Senior/ Geriatric	MC	12.6	17 (37.4)	12.57	1.00	Mixed breed
S5	Senior/ Geriatric	MC	13.8	10.3 (22.7)	13.59	1.02	Jack Russell terrier (12-14y)
S6	Senior/ Geriatric	MC	13.4	28.2 (62.0)	11.85	1.13	Mixed breed
S7	Senior/ Geriatric	FS	11.4	36.9 (81.8)	11.64	0.98	Irish Setter (12-15y)
S8	Senior/ Geriatric	MC	12.4	31.6 (62.5)	11.64	1.07	Golden retriever (10-12y)

S9	Senior/ Geriatric	MC	11.9	20.6 (45.3)	12.86	0.93	German Shorthair pointer (10-12y)
S10	Senior/ Geriatric	MC	12.3	23.5 (51.7)	12.14	1.01	Golden Retriever (10-12y)
S11	Senior/ Geriatric	FS	11.8	18.9 (41.6)	12.35	0.96	Basset Hound (12-13y)
S12	Senior/ Geriatric	FS	12.8	22.4 (49.3)	12.43	1.03	Am. Staff (12-16y)
S13	Senior/ Geriatric	FS	11.7	10.8 (23.8)	13.44	0.87	Mixed Breed
S14	Senior/ Geriatric	FS	14.8	11.1 (24.4)	13.46	1.10	Mixed breed
S15	Senior/ Geriatric	FS	14.4	21 (46.2)	12.79	1.13	Brittany spaniel (12-14y)
S16	Senior/ Geriatric	FS	13.3	4.3 (9.5)	14.06	0.95	Pomeranian (12-16y)
S17	Senior/ Geriatric	FS	13.3	26.7 (58.7)	12.23	1.09	Siberian Husky (12-14y)
S18	Senior/ Geriatric	FS	14.5	7.5 (16.5)	13.64	1.06	Mixed breed
